# Development and validation of ‘Patient Optimizer’ (POP) algorithms for predicting surgical risk with machine learning

**DOI:** 10.1186/s12911-024-02463-w

**Published:** 2024-03-11

**Authors:** Gideon Kowadlo, Yoel Mittelberg, Milad Ghomlaghi, Daniel K. Stiglitz, Kartik Kishore, Ranjan Guha, Justin Nazareth, Laurence Weinberg

**Affiliations:** 1 Atidia Health, Melbourne, Australia; 2 Department of Anaesthesiology and Perioperative Medicine, Alfred Health, Melbourne, Australia; 3 Data Analytics Research and Evaluation Centre, Austin Health, Melbourne, Australia; 4 Department of Anaesthesia, Austin Health, Heidelberg, Australia; 5 Department of Critical Care, The University of Melbourne, Austin Health, Heidelberg, Australia

**Keywords:** Post-operative complications, Pre-operative care, Risk prediction, Risk assessment, Machine learning

## Abstract

**Background:**

Pre-operative risk assessment can help clinicians prepare patients for surgery, reducing the risk of perioperative complications, length of hospital stay, readmission and mortality. Further, it can facilitate collaborative decision-making and operational planning.

**Objective:**

To develop effective pre-operative risk assessment algorithms (referred to as Patient Optimizer or POP) using Machine Learning (ML) that predict the development of post-operative complications and provide pilot data to inform the design of a larger prospective study.

**Methods:**

After institutional ethics approval, we developed a base model that encapsulates the standard manual approach of combining patient-risk and procedure-risk. In an automated process, additional variables were included and tested with 10-fold cross-validation, and the best performing features were selected. The models were evaluated and confidence intervals calculated using bootstrapping. Clinical expertise was used to restrict the cardinality of categorical variables (e.g. pathology results) by including the most clinically relevant values. The models were created with logistic regression (LR) and extreme gradient-boosted trees using XGBoost (Chen and Guestrin, 2016). We evaluated performance using the area under the receiver operating characteristic curve (AUROC) and the area under the precision-recall curve (AUPRC). Data was obtained from a metropolitan university teaching hospital from January 2015 to July 2020. Data collection was restricted to adult patients undergoing elective surgery.

**Results:**

A total of 11,475 adult admissions were included. The performance of XGBoost and LR was very similar across endpoints and metrics. For predicting the risk of any post-operative complication, kidney failure and length-of-stay (LOS), POP with XGBoost achieved an AUROC (95%CI) of 0.755 (0.744, 0.767), 0.869 (0.846, 0.891) and 0.841 (0.833, 0.847) respectively and AUPRC of 0.651 (0.632, 0.669), 0.336 (0.282, 0.390) and 0.741 (0.729, 0.753) respectively. For 30-day readmission and in-patient mortality, POP with XGBoost achieved an AUROC (95%CI) of 0.610 (0.587, 0.635) and 0.866 (0.777, 0.943) respectively and AUPRC of 0.116 (0.104, 0.132) and 0.031 (0.015, 0.072) respectively.

**Conclusion:**

The POP algorithms effectively predicted any post-operative complication, kidney failure and LOS in the sample population. A larger study is justified to improve the algorithm to better predict complications and length of hospital stay. A larger dataset may also improve the prediction of additional specific complications, readmission and mortality.

**Supplementary Information:**

The online version contains supplementary material available at 10.1186/s12911-024-02463-w.

## Introduction

The adoption and deployment of electronic health records (EHRs) has facilitated the accessibility of large patient datasets. Machine learning (ML) has succeeded in diverse arenas, demonstrating an ability to operate on large and complex datasets. At the intersection of EHR data and the progress of ML, is an opportunity to develop tools for personalised medicine. Currently, the most common ML applications in medicine are in imaging [1, 2]. An upcoming frontier is surgical risk prediction [3].

Surgery is often the only option to alleviate disability and reduce the risk of death from common conditions. Millions of people annually undergo surgical treatment, and surgical interventions account for an estimated 13% of the world’s total disability-adjusted life years (DALYs). Even in the most advanced hospital systems, there is a high mortality and complication rate [4, 5], risks of direct harm to patients and high financial costs. The WHO recognises these issues as major worldwide health burdens [6]. Fortunately, a substantial number of these complications are preventable [7].

Pre-operative risk assessment allows clinicians to mitigate adverse outcomes, better inform patients and their families about surgical outcomes and risks and plan post-operative care [8, 9]. The first generation of risk calculators exists, such as the American College of Surgeons National Surgical Quality Improvement Program (NSQIP) [10] and the Surgical Outcome Risk Tool (SORT) [11]. They are based on linear statistical techniques and are designed to use a low number of input parameters to be convenient for manual data entry. These approaches do not exploit the data available in modern EHR systems. Additionally, most provide mortality risk only. There are also manual risk assessments such as American Society of Anesthesiologists (ASA) Physical Status Classification [12] that are effective but subjective. It is often difficult for clinicians to find the data and calculate the score manually; therefore, they are rarely used [13].

In recent years, more sophisticated algorithms have been developed using ML. They typically predict a wider range of outcomes than traditional risk calculators and incorporate a larger set of input features made available by EHR data. The most common prediction outcomes are mortality and post-surgical complications such as acute kidney injury, delirium, deep vein thrombosis, pulmonary embolism and pneumonia. ML can be more effective than traditional methods [13] such as ASA, CCI, POSSUM [14] and NSQIP [15] and can be more effective than human experts [16]. Various techniques have been used such as deep learning [17, 18], logistic regression [19, 20], generalised additive models (GAMs) [5] and decision trees [15, 21–25]. Further, some studies focus on harmonising EHR data [17], testing existing approaches on suitability for local populations [3, 19] or predicting the use of the readmission prevention clinic [21].

Most of the studies in the literature cited above, focus on the prediction of mortality and complications; however, additional endpoints are clinically meaningful. Some studies such as [17, 25], utilise sequences of vital sign measurements, unstructured notes and radiological images, but in many hospitals, those data are not practically obtainable.

### Study aims

This study aims to use readily available EHR data to develop interpretable ML risk prediction algorithms to standardise and improve clinical decision-making. The target endpoints are length-of-stay (LOS), complications, unplanned 30-day readmission and in-patient mortality. Our definition of readily available EHR data is patient history, excluding unstructured notes and radiological imaging. The algorithms should be interpretable as the ultimate objective is to provide information that is understandable, actionable and trusted in a clinical setting.

## Method

### Study design

This was a single-centre cohort study with retrospective data collection in adult patients (aged $\ge 18$ years) who underwent any elective surgical procedure at Austin Health between 1st January 2015 and 31st July 2020. Austin Health is a university teaching hospital in Australia, with a high volume of surgeries across multiple sub-specialities that are performed annually. We restricted cases to elective surgery, which comprises $\sim$85% of surgical cases in Australia [26] and where there is the greatest opportunity to mitigate risk, based on perioperative risk prediction. Currently risk predictors such as ASA and NSQIP are standard tools used by perioperative physicians to assess risk in elective surgery patients. First and foremost, elective surgery affords time for a thorough pre-operative evaluation and optimisation of the patient and the opportunity to choose many factors that influence their care, such as theatre location (ICU availability), blood availability, and many others. In addition, predictions have operational utility, for example for planning and scheduling to ensure higher patient throughput.

We developed risk assessment models for the target endpoints following the Transparent Reporting of a Multivariable Prediction Model for Individual Prognosis or Diagnosis (TRIPOD) guidelines for risk prediction [27].

Our approach was to begin with a base model that emulates a standard approach internationally for surgical risk assessment, exploiting two key dimensions: patient-risk and procedure-risk. Each was derived from clinical expertise provided by perioperative clinicians with at least 10-years of postgraduate experience and familiarity with risk stratification for surgical morbidity. The next step was to iteratively add features to the base model, resulting in a unique set of features for each model.

### Model development

We evaluated our method with two model types. First, logistic regression (LR) (using scikit-learn [28]), a widely accepted and relatively straightforward linear approach, which served as a baseline. Second, we compared to extreme gradient-boosted decision trees (XGBoost, using the XGBoost package [29]), a more complex algorithm that is capable of capturing non-linear decision boundaries and interactions between features. XGBoost models are interpretable and among the best performing for tabular data.

**Fig. 1:**
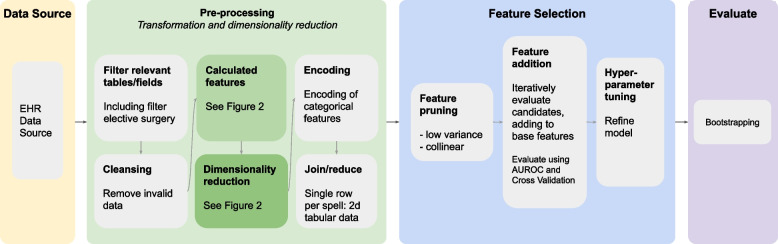
Data processing pipeline

There are four main stages to the method. The **Data source** provides data for **Pre-processing** that reduces dimensionality and transforms relational data into a tabular form suitable for algorithm consumption. **Feature selection** selects a subset of features to optimise prediction scores for each endpoint. Finally, an **Evaluation** of the models is performed with bootstrapping. The pipeline is illustrated in Fig. 1 and elaborated below.

#### Data source

The Data Analytics and Research Evaluation (DARE) Centre provided a data extract from the Austin Health Cerner EHR system. A total of 11,475 unique admissions were included, covering all elective adult surgical procedures.

The raw data includes:

- patient demographic details (age, weight, height, gender)
- procedures performed (primary/scheduled and other)
- other procedural information (including details of the admission and episode)
- pathology results
- medications prescribed during admission
- comorbidities: diagnoses using the International Classification of Diseases (ICD-10-AM, 9th Edition)
- Charlson comorbidity index (CCI) derived from the ICD codes
- complications, indicated by ICD codes

#### Pre-processing

**Fig. 2:**
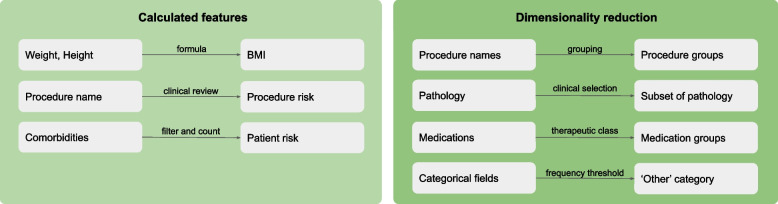
Dimensionality reduction and calculated features

After filtering and cleansing the data, we derived features from raw values: body mass index (BMI) and the two features used for the base model, namely estimates of patient-risk and procedure-risk (“Study design” section). The process is illustrated in Fig. 2.

Patient-risk is a proxy for ASA [12]. It is an ordinal numerical value calculated through the patient’s diagnoses (using ICD codes). Each ICD code was scored by a clinician as either included or excluded from patient risk (score of 0 or 1). In addition, codes that represented ‘cancerous’ or ‘cardiopulmonary and vascular’ were scored higher (score of 2). The total of the ICD risk scores attributable to a patient admission was used to calculate the patient-risk.

Procedure-risk is an ordinal categorical value calculated using a clinically determined risk rating (low, medium or high) of the scheduled surgical procedure, which was estimated as the earliest non-preparatory procedure.

We reduced dimensionality where possible to reduce overfitting and improve interpretability. We grouped procedure names by anatomical region. Although all patient episodes are elective, the individual procedures can be varying levels of elective or emergency, referred to as procedure type. The procedure type was reduced to a binary category. For laboratory results, we selected *a priori* eight clinically relevant variables, namely haemoglobin, albumin, creatinine, urea, international normalised ration, platelet count, activated partial thromboplastin time, and estimated glomerular filtration rate. We grouped patient medications by therapeutic class. Finally, very infrequent categories were grouped into an ‘other’ bucket. Dimensionality reduction is summarised in Fig. 2.

Categorical data was one-hot encoded. Where there were one-to-many relationships (such as admission to medications) the reduction methods were chosen to provide the most clinically relevant summary.

Missing categorical data were treated as legitimate input by creating a ‘missing’ category. Missing numerical data were imputed with XGBoost’s in-built mechanism and by using the median for LR. For LR, numerical data was standardized (using scikit-learn’s StandardScaler). Class balancing was achieved by applying a higher weight to under-represented classes.

See Supplementary Materials, Section 1 for more details on pre-processing.

#### Feature selection

Highly correlated (or collinear) features were removed due to their redundancy. We used the variance inflation factor method for multi-collinearity analysis with a threshold of 10 [30, 31]. Variables with very low variance were removed by detecting features where the ratio between the highest occurring value and the second highest was greater than 19, a large threshold to avoid losing valuable information [32].

After training and scoring the base model consisting of patient-risk and procedure-risk (“Study design” section), an automated iterative process added and tested new features. Each available feature was individually added to the model and evaluated using area under the receiver operating characteristic curve (AUROC) with 10-fold cross-validation. The feature that achieved the highest gain in score was added to the selected feature set and the search restarted. The remaining features were re-tested in subsequent iterations, after which the composition of the selected feature-set had changed. The process continued until all features were used and then the model with the highest score was selected.

Hyperparameter tuning then took place to optimise results (see Supplementary Materials, Section 2).

### Predicted outcomes

**Fig. 3:**
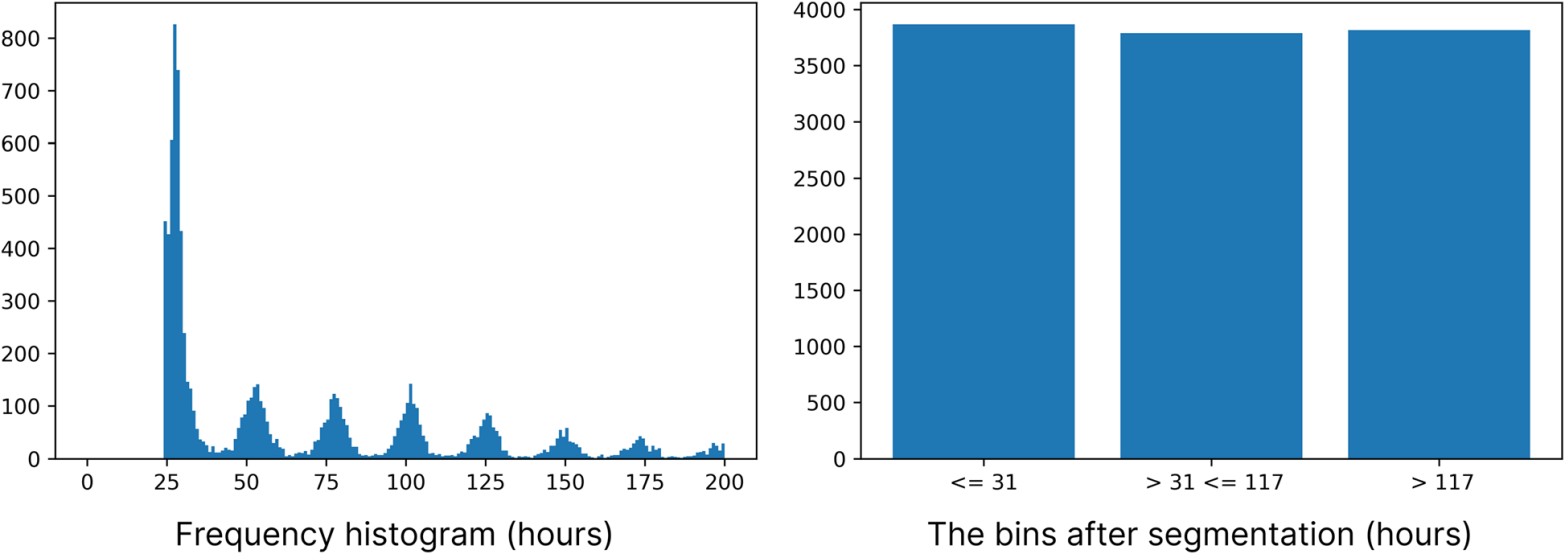
Length-of-stay: Training data are segmented into 3 classes, to cast predicting length-of-stay as multiclass classification. There is a clear periodicity around whole days. The x-axis is truncated at 200 hours to provide detail in the most interesting range. The trend continues past 200 hours with a steady monotonic decrease in magnitude

Length-of-stay (LOS) was framed as multiclass classification. We identified three dominant groupings through visual inspection of the distribution (see Fig. 3) and defined them by ordering and then segmenting the data into three equally sized buckets. The resulting groups were ‘low’ ($\le$31 hours), ‘medium’ (31 – 117 hours), and ‘high’ ($\ge$117 hours), equating to one night, two to four nights and five nights or more. The ranges were validated through clinical review. There was a classifier for each bucket and the prediction was the classifier with the highest confidence. The labels (low, medium and high) do not describe clinical significance, which depends on the procedure type. For example, ‘medium’ duration may be considered prolonged for a simple procedure, whereas ‘medium’ may be expected for a more complex procedure.

Unplanned 30-day readmission (hereafter abbreviated to ‘30-day readmission’), in-patient mortality and the presence of complications (as indicated by the ICD code) were explicitly labelled in the dataset.

### Evaluation

The final score and confidence intervals were calculated with non-parametric bootstrapping using 1,000 iterations. For each iteration the training set size was the same as the whole dataset. As bootstrapping involves sampling with replacement, this resulted in approximately 70% unique samples for training, leaving the left-out 30% for testing.

#### Performance metrics

Several metrics were used to assess and measure performance: area under the receiver operating characteristic curve (AUROC), area under the precision-recall curve (AUPRC) and F1 (FBeta, where beta = 1). AUROC is most common in related literature. A drawback of AUROC is that it can be misleading on extremely rare classes such as mortality and readmission. In such cases, it can achieve an artificially high score because the true negatives dwarf the false positives^1^. AUPRC is more informative with extremely rare labels [14, 33]. It indicates the trade-off between precision (the proportion of true positives of all predicted positives), also referred to as positive predictive value (PPV), and recall (the proportion of true positives of all positives). F1, the harmonic mean of precision and recall, is also suitable for rare classes. We used micro-averaging to calculate the area under the curve for multiclass predictions.

In addition to the single metric derived from the ‘area’ under the respective curves AUROC and AUPRC, we also inspected the profiles of the curves, showing how they perform at different operating points.

#### Interpretability

To visualise the relative importance of features for each model, we utilised two methods. The first was XGBoost feature importance, based on the average gain of splits per feature. The other was SHapley Additive exPlanations (SHAP) [34] which uses cooperative game theory to assign partial credit to the input variables for the model’s output. Both methods indicate feature importance from different perspectives. XGBoost feature importance gives direct insight into the internal structure of the learned trees and provides a single absolute value for importance. SHAP treats the model as a black box and bases the importance on the observed behaviour of the model. The plots are more informative, showing the distribution of observed values and the corresponding directionality of the impact on the model.

To visualise the features’ influence on specific predictions for individual patients, we used SHAP. We plotted typical true positives for each of the effective models to demonstrate how SHAP can be used to help make specific predictions actionable.

The predictions combined with visualisations could allow clinicians to understand the most important features in general, while providing per-patient feedback on the key features contributing to a prediction. Consequently, clinicians can take appropriate actions to address patient or procedure factors to minimise risk.

In addition, the predictions together with visualisations could enable hospitals to improve decision making, such as pre-admission patient optimization or capacity planning i.e. booking theatres or hospital resources. For example, if a patient is expected to have a longer than expected length-of-stay, the hospital administration could anticipate that they would take up a hospital bed for a period longer than typical for the relevant procedure.

## Results

### Data characteristics

A total of 11,475 adults were included. There were 41 (0.36%) occurrences of in-patient mortality and 941 (8.2%) occurrences of 30-day readmissions. There were 4,351 (37.92%) complications. The number of occurrences of low, medium and high LOS were 3,868 (33.7%), 3,790 (33.0%) and 3,817 (33.3%), respectively. The data characteristics are presented in Table 1.

**Table 1:** Data characteristics: The first column shows the number of affirmative cases for binary fields and the number of unique values for multivalue categorical fields. The second column shows the number of admissions with a valid value (e.g., if height is missing, it is deemed invalid). Empty cells denote N/A

	# Affirmative / # Categories	# Valid values	Median	Mean (SD)
**Demographics**				
M	6,234 (54.33%)	11,475 (100.00%)		
F	5,241 (45.67%)	11,475 (100.00%)		
Age		11,475 (100.00%)	62.00	59.24 (17.81)
Height		4,958 (43.21%)	167.00	166.01 (14.63)
Weight		6,920 (60.31%)	81.00	84.13 (21.46)
**Derived features**				
BMI		4,931 (42.97%)	29.38	32.10 (14.09)
Procedure-risk		11,475 (100.00%)	1.00	1.63 (0.71)
Patient-risk		11,475 (100.00%)	3.00	4.06 (3.13)
**Other**				
Emergency procedure	1,416 (12.34%)	11,475 (100.00%)		
**Categorical fields**				
Procedures	911			
Medication	923			
Pathology	75			

### Accuracy

**Fig. 4:**
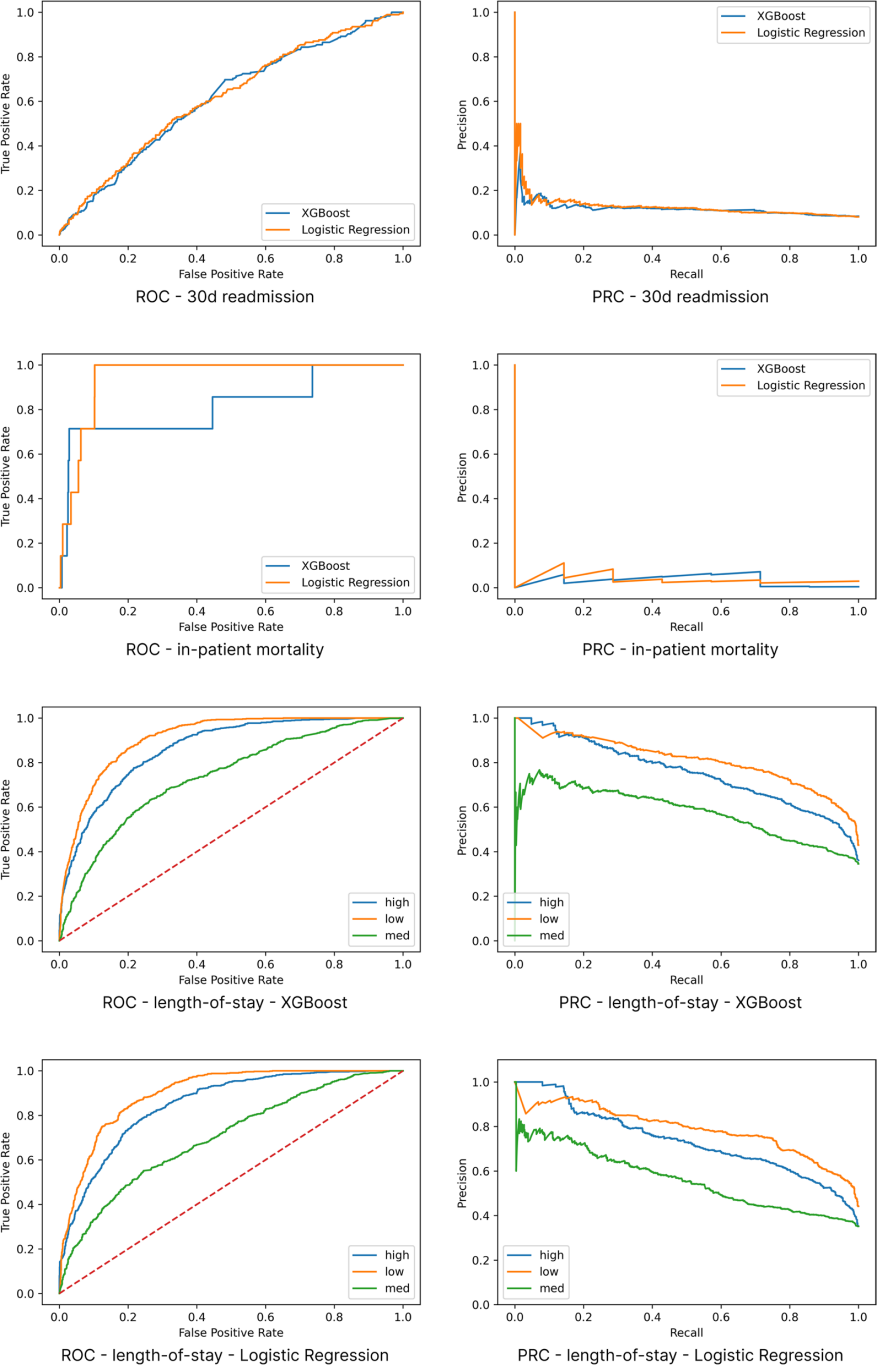
Receiver operating characteristic and precision-recall curves – readmission, mortality and length-of-stay. The mortality curves appear stepped due to the fact that there are only 7 positive samples

**Fig. 5:**
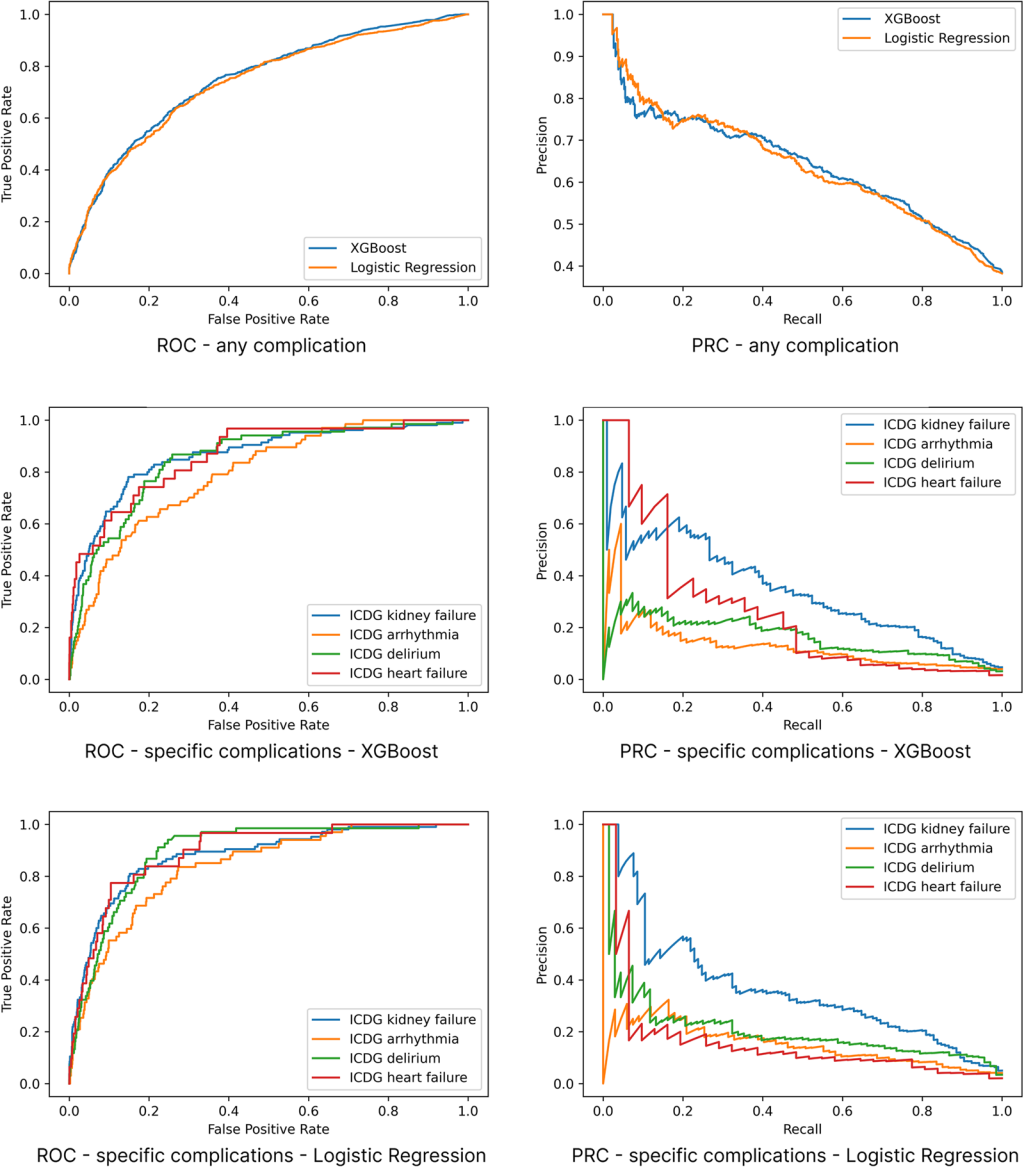
Receiver operating characteristic and precision-recall curves – complications. The mortality curves appear stepped due to the fact that there are only 7 positive samples

The results are summarised in Tables 2 and 3, and the ROC and PR curves are shown in Figs. 4 and 5. We selected only those specific complications with an adequate number of positive examples to make training feasible (above a threshold of 100 (0.8%), including kidney failure, arrhythmia, delirium and heart failure. Other complications such as cardiac or respiratory arrest, liver failure, pulmonary embolism, and respiratory failure did not have sufficient data points.

**Table 2:** Performance of risk models

Prediction	# Cases (prevalence)	Model	AUROC (95% CI)	AUPRC (95% CI)	F1 Value (95% CI)
In-patient mortality	41 (0.36%)	XGBoost	0.866 (0.777, 0.943)	0.031 (0.015, 0.072)	0.057 (0.000, 0.167)
		Logistic regression	0.914 (0.811, 0.956)	0.044 (0.019, 0.114)	0.044 (0.028, 0.063)
30-day readmission	941 (8.20%)	XGBoost	0.610 (0.587, 0.635)	0.116 (0.104, 0.132)	0.122 (0.078, 0.156)
		Logistic regression	0.622 (0.599, 0.645)	0.130 (0.113, 0.149)	0.189 (0.171, 0.206)
Length-of-stay	N/A	XGBoost	0.841 (0.833, 0.847)	0.741 (0.729, 0.753)	0.666 (0.654, 0.678)
		Logistic regression	0.822 (0.815, 0.829)	0.719 (0.707, 0.730)	0.646 (0.634, 0.658)

**Table 3:** Performance of risk models for complications

Prediction	# Cases (prevalence)	Model	AUROC (95% CI)	AUPRC (95% CI)	F1 Value (95% CI)
Any complication	4,351 (37.92%)	XGBoost	0.755 (0.744, 0.767)	0.651 (0.632, 0.669)	0.621 (0.602, 0.639)
		Logistic regression	0.747 (0.735, 0.760)	0.646 (0.628, 0.665)	0.629 (0.615, 0.644)
Heart failure	116 (1.01%)	XGBoost	0.835 (0.773, 0.887)	0.101 (0.055, 0.181)	0.141 (0.097, 0.190)
		Logistic regression	0.878 (0.834, 0.915)	0.101 (0.058, 0.184)	0.087 (0.071, 0.104)
Delirium	303 (2.64%)	XGBoost	0.827 (0.793, 0.857)	0.139 (0.099, 0.187)	0.189 (0.153, 0.225)
		Logistic regression	0.873 (0.851, 0.896)	0.181 (0.134, 0.233)	0.169 (0.150, 0.187)
Arrhythmia	341 (2.97%)	XGBoost	0.794 (0.764, 0.822)	0.122 (0.092, 0.165)	0.148 (0.129, 0.169)
		Logistic regression	0.831 (0.800, 0.859)	0.156 (0.121, 0.204)	0.155 (0.138, 0.171)
Kidney failure	505 (4.40%)	XGBoost	0.869 (0.846, 0.891)	0.336 (0.282, 0.390)	0.326 (0.293, 0.359)
		Logistic regression	0.883 (0.863, 0.901)	0.308 (0.258, 0.363)	0.285 (0.262, 0.309)

For predicting the risk of any post-operative complication, kidney failure and LOS, XGBoost achieved an AUROC (95%CI) of 0.755 (0.744, 0.767), 0.869 (0.846, 0.891) and 0.841 (0.833, 0.847) respectively and AUPRC of 0.651 (0.632, 0.669), 0.336 (0.282, 0.390) and 0.741 (0.729, 0.753), respectively; LR achieved an AUROC (95%CI) of 0.747 (0.735, 0.76), 0.883 (0.863, 0.901) and 0.822 (0.815, 0.829) respectively and AUPRC of 0.646 (0.628, 0.665), 0.308 (0.258, 0.363) and 0.719 (0.707, 0.73), respectively. Refer to the table for full results of other specific complications.

For 30-day readmission and in-patient mortality, XGBoost achieved an AUROC (95%CI) of 0.61 (0.587, 0.635) and 0.866 (0.777, 0.943), respectively and AUPRC of 0.116 (0.104, 0.132) and 0.031 (0.015, 0.072), respectively; LR achieved an AUROC (95%CI) of 0.622 (0.599, 0.645) and 0.914 (0.811, 0.956), respectively and AUPRC of 0.13 (0.113, 0.149) and 0.044 (0.019, 0.114), respectively.

On visual inspection, the ROC curves provide reasonable operating points for all models. Inspection of the precision-recall (PR) curves also shows some models have effective operating points; although the endpoints with extremely rare positive examples do not, including readmission, mortality, and the specific complications other than kidney failure. For LOS, accuracy was consistently higher for the two ends of the spectrum (low and high) compared to medium which experienced more class overlap than low or high.

The performance of XGBoost was very similar to LR, and there was no clear winner across metrics or endpoints.

### Interpretability

**Fig. 6:**
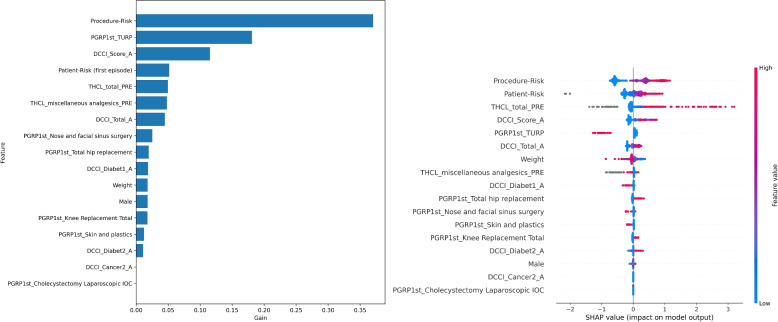
Feature importance for any complication: XGBoost gain (left) and SHAP (right), where each dot represents one sample, the colour indicates the value and the position on the x-axis indicates the impact (positive or negative) on model output. Refer to Table 4 for terminology

**Fig. 7:**
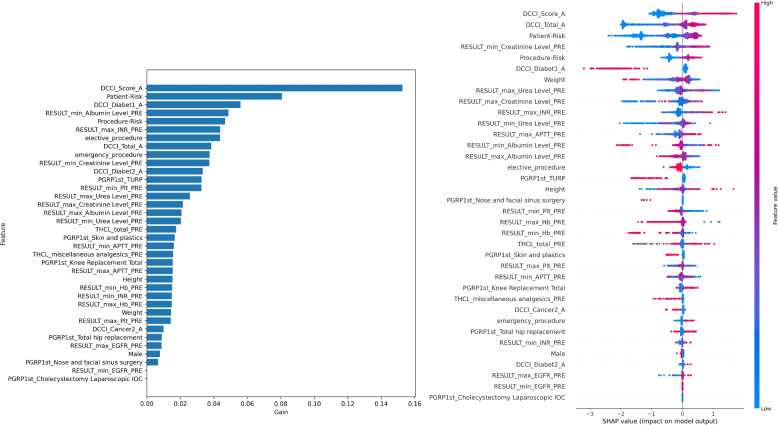
Feature importance for kidney failure: XGBoost gain (left) and SHAP (right), where each dot represents one sample, the colour indicates the value and the position on the x-axis indicates the impact (positive or negative) on model output. Refer to Table 4 for terminology

**Fig. 8:**
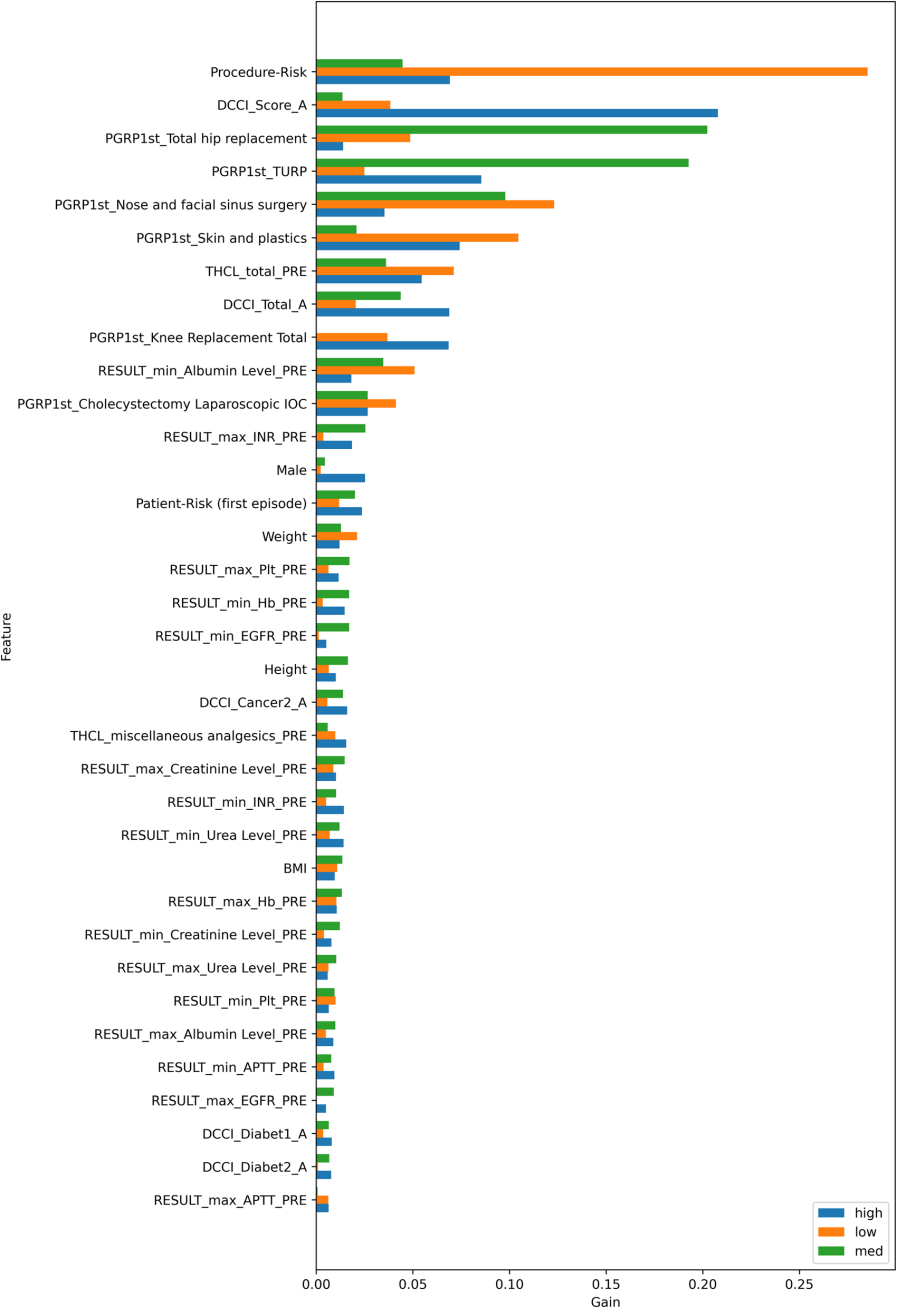
Feature importance for length-of-stay using XGBoost gain. Refer to Table 4 for terminology

For simplicity, we used one model type to explore interpretability. We chose XGBoost, as the performance of XGBoost and LR was comparable, and XGBoost is capable of finding more complex relationships which may be relevant for other datasets. The selected features and their importance are shown for the effective models: complications in Fig. 6, kidney failure in Fig. 7 and LOS in Fig. 8. For terminology used in the figures, please refer to Table 4.

**Table 4:** Feature-name terminology

Term	Meaning
DCCI	Diagnosis via Charlson Comorbidity Index
DCCI_Score_A	Age adjusted DCCI score [35] at admission
DCCI_Total_A	Raw total DCCI score at admission
PGRP	Procedure group
RESULT	Pathology test result
THCL	Medication (therapeutic class)

For all the models and visualisation methods, procedure-risk and features representing the patient’s health (CCI summaries and patient-risk) are amongst the top factors. Patient-risk and CCI represent the patient’s overall health. Although patient-risk is derived from more specific and diverse comorbidities than CCI, the feature importance plots showed that across the cohort, CCI was an important factor particularly in the age-adjusted CCI [35], and comparable to patient-risk. However, patient-risk and CCI are both valuable as they contain different information, as illustrated in the example of a specific patient high LOS, Fig. 10, where patient-risk and CCI have an opposing influence.

In addition to procedure-risk and patient health, there are other important features. For any complication (Fig. 6), XGBoost shows significant tree splits for some specific procedure groups: diabetes, total medication dosages and use of analgesics. The SHAP features are largely aligned, with differences in the relative values. For kidney failure (Fig. 7), related morbidities (diabetes, cancer) and pathology results (albumin, creatinine, urea, activated partial thromboplastin time and haemoglobin) are also important. For length-of-stay (Fig. 8), the features had differing importance to the individual models (low, medium and high), although many features are unimportant for all models. Compared to the other models, specific procedure groups are relatively important.

**Fig. 9:**
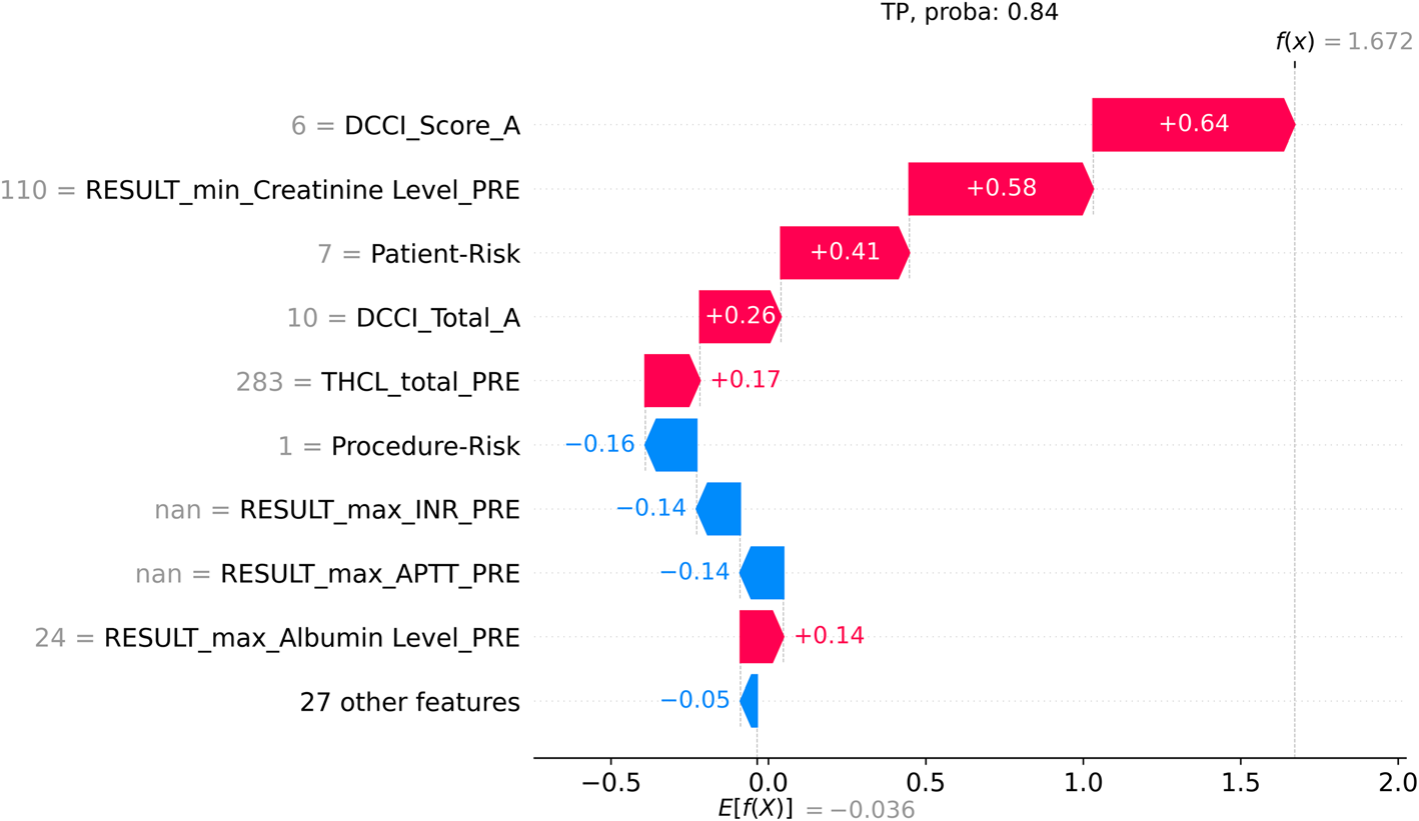
SHAP visualisation for a specific patient’s risk of kidney failure. This is a True Positive (TP) prediction with a probability of 0.87. The length of the bar indicates the influence of that feature on the prediction. The colour indicates whether the influence is positive (red) or negative (blue). The grey value to the left of the feature name is the value of that feature for this patient

**Fig. 10:**
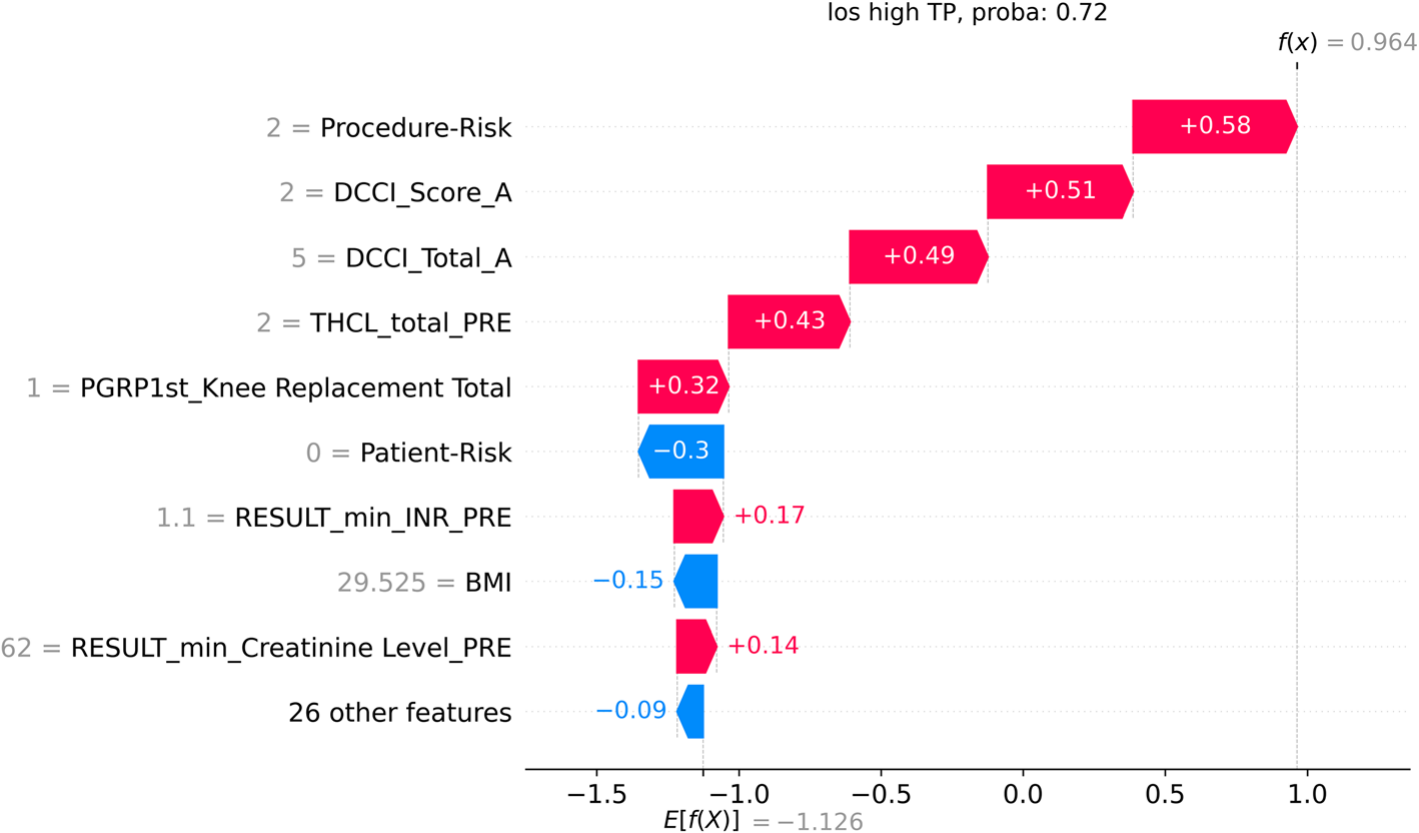
SHAP visualisation for a specific patient’s length-of-stay. This is a True Positive (TP) prediction of a high length-of-stay, with a probability of 0.72. The length of the bar indicates the influence of that feature on the prediction. The colour indicates whether the influence is positive (red) or negative (blue). The grey value to the left of the feature name is the value of that feature for this patient

Feature importance in specific predictions using SHAP plots is shown for correct predictions of a) kidney failure (Fig. 9) and b) ‘high’ LOS for a procedure that typically has a medium-term LOS (Fig. 10). The purpose is to show how SHAP can provide a convenient interpretation of the important factors for a given prediction.

## Discussion

### Key findings

In this single-centre cohort study in adult surgical patients, we developed effective pre-operative risk assessment algorithms (POP) using machine learning, providing pilot data to inform the design of a larger prospective study. We found that POP algorithms were effective for predicting post-operative complications and LOS. However, a larger study is justified to further improve the algorithm for predicting specific complications, readmission and mortality.

The performance of logistic regression (LR) and XGBoost models was similar, with no clear winner across endpoints and metrics; suggesting that for this set of features, a linear decision boundary is sufficient and there are no significant relationships between features. It is possible that with more patients and/or more features (such as patient notes and imaging), non-linear methods such as XGBoost would outperform LR. For simplicity, we focus on one model type, XGBoost, in the interpretability results and for the remainder of the Discussion. We chose XGBoost as it is more capable and has superior ease-of-use.

### Comparison to other methods

Comparing accuracy to other models in the literature is very difficult for several reasons. The quality and structure of different datasets vary greatly, cohort differences can influence results [36] and endpoints are often defined differently (e.g., 24 hours after admission compared to immediately before surgery). Moreover, the choice of performance metrics also varies. However, considering the difficulties, it can be useful to compare results to provide some context.

One of the studies that we compared to is Rajkomar et al. [17]. Beyond tabular EHR data, they used additional data sources including radiological imaging, unstructured notes, vital sign measurements, time-series embedding to handle these data streams, as well as ensembling of complementary models. While we consider it to be the ‘gold-standard’, and therefore present it as context, we do not aim to match their scores. Our study investigates the feasibility of risk predictions with more limited and commonly available data sets.

In studies with similar objectives to ours, authors compared LR and variations of Boosted Decision Trees such as XGBoost (as well as other algorithms) [14, 15, 25]. In general, the Boosted Decision Tree algorithms were superior to LR, although similarly to our results, Corey et al. [15] found LR and XGBoost had very similar performance. Where Boosted Decision Trees had an advantage, it could be due to the difference in available features or in the methodology used in feature selection. The model development pipeline in our study selected the most suitable features for each algorithm, and as a result they may use different features to predict any particular label. In addition, there were differences in the set of predicted outcomes; compared to mortality, ICU admission, and complications [14, 15, 25] our successful models also included length-of-stay. Regardless of which algorithm performed the best, these studies supported our findings that Machine Learning models can provide useful – and in the case of [25], interpretable – predictions, to assist clinical decision making.

### Comparison to standard risk predictors

Standard risk predictors, such as ASA or CCI provide one score that captures patient risk. Such a score can be used to predict ‘general’ outcomes, such as the use of ASA to predict mortality and ICU Admission, but has been found to be less effective than ML approaches [14]. Moreover, it is not clear how to directly translate the score into more specific outcomes such as length-of-stay or specific complications. Such outcomes could provide additional administrative or clinical insights to assist with patient management and decision making.

However, standard risk predictors can be used as features, as we did in this study. Feature importance analysis showed that CCI and patient-risk (a proxy metric for ASA) are indeed significant contributors to model prediction. Other features, such as pathology results or medication classes, are also identified by the model as adding predictive value.

### Evaluation metrics

The standard practice for evaluating risk prediction algorithms is the ROC curve. Using ROC, all of our models appear to be effective. They have a good profile with viable operating points, and relatively good AUROC. However the results using the PR curves reveal a different story. AUPRC for readmission and mortality is very low, and there are no satisfactory operating points on the profile. The results confirm that ROC can be misleading for rare classes as suggested by [14] (and discussed in “Evaluation” section). They demonstrate the importance of metrics that are insensitive to rare classes, such as AUPRC or FBeta for clinical algorithms. We used a relatively small dataset (see “Limitations” section). With more data and therefore more positive examples, the performance is likely to improve, as measured by both AUROC and AUPRC.

### LOS prediction

LOS classification was very effective. There is LOS data for every admission, providing ample training signal, which is reflected in the ROC and PR curves. LOS predictions have both clinical and operational decision making benefits. From a clinical perspective, a prolonged or ‘longer than expected’ stay prediction could prompt closer attention. From an operational perspective, these predictions could be used for scheduling to optimise for ward utilisation and selection of appropriate sites.

To the best of our knowledge, other ML risk predictors did not consider LOS, except [17]. They predicted ‘prolonged length-of-stay’, defined as ‘at least 7 days’, whereas POP predicts multivalue LOS: low, medium or high. Predicting multivalue LOS makes it possible to have a dynamic definition of ‘prolonged’ that depends on factors such as procedure and patient. For example, a medium stay (two to four nights) prediction could trigger ‘prolonged’ for short-stay surgery (1 night) and healthy patients. Secondly, a more granular prediction allows better operational planning. Our accuracy, measured using AUROC, was comparable to [17], 0.841 compared to 0.85 and 0.86 (for two hospital sites respectively), despite fewer data types and a much smaller dataset. Unfortunately AUPRC was unavailable for comparison to gain a fuller picture.

### Complication prediction

Results for predicting any complication were promising, with both AUROC and AUPRC having viable operating points. Of all the complications, four had adequate positive examples to train the models. These had reasonable ROC curves, but precision and recall showed that only kidney failure, which is less rare than the others, was a viable model.

In a clinical setting, positive predictions could be used as a general indicator that there is morbidity, and investigations are warranted. An example of an operating point for kidney failure is approximate recall of 12%, and precision of 62%. Out of 100 patients with kidney failure, the model will identify approximately 12. Of those, approximately 62% (7.4) will actually develop kidney failure (true positives). If the information is presented so that it doesn’t give a false sense of security if *not* shown, then it can pick up when there *is* a case, aiding clinical care.

The results compare favourably to similar studies, despite a much smaller dataset (“Limitations” section). Across specific complications, and using AUROC, POP XGBoost models scored 0.794 – 0.869 compared to 0.820 – 0.940 [5], 0.772 – 0.909 [15] and 0.88 – 0.89 [17]. For any complication, POP XGBoost scored 0.755 compared to 0.829 – 0.836 in [15]. Again, PR results are unavailable for a more complete comparison. Precision (referred to as PPV or positive predictive value) was reported in [5], which showed the same pattern as POP with rare classes (i.e., the rarer classes generally have lower precision).

### Interpretability

The introduction of ML often leads to improved performance, but it can come at the cost of interpretability. We used XGBoost and SHAP feature importance plots. They are intuitive and build trust in the model, helping to make it understandable and actionable.

The relative importance of features learned by the algorithm aligns with clinical practice. For example, the high importance of procedure information combined with patient health is commonly used to assess the risk of surgery. Alignment with clinical practice provides confidence that learning is effective and generalisable. Additionally, the relative weighting of feature importances can provide new insights into the relationship between features and outcomes. Although not causative, it indicates a relationship, and warrants further investigation. A better understanding of the factors, especially modifiable ones, could impact clinical practice.

The first type of visualisation is the feature importance of the model in general, indicating systematic relationships across samples in the dataset. The other type of visualisation was feature importance for specific predictions, which highlights factors for individual patients. This information can provide an opportunity for more personalised risk mitigation.

We now explore kidney failure as a case study. The model highlighted comorbidities (Fig. 7) that align with current knowledge. Several pathology results are also considered important; for example, some known to be related to renal function such as creatinine and urea, and others that are generally indicative of post-operative outcomes such as albumin [4], pathology related to coagulation (INR, APTT, PLT) [37, 38] and heamaglobin (Hb) [39, 40]. Some procedure groups were protective: ‘trans-urethral resection of the prostate’ (TURP), likely because it improves renal function; and ‘nose and facial sinus surgery’, likely because it is very low risk. The importance of ‘total knee replacement’ is unexpected, and warrants further investigation; for example, the underlying cause may be tourniquet time, length of surgery or even anaesthetic type.

Surprisingly, diabetes is protective. We hypothesise that patients with this condition are more actively managed, so it is not picked up by the model, which learns from raw correlations. Alternatively, it could be due to conflating factors, which may have a higher than usual impact on results due to the small number of positive samples. It is also the likely explanation for a similarly protective effect in the model for any complication (Fig. 6). It would be beneficial to repeat the study after gathering a larger sample size, and a more thorough investigation that includes causal analysis is an important topic for future work.

Understanding the expected and unexpected features may allow for patient-specific pre-operative intervention to minimise post-operative complications. For example, by optimising HbA1c in diabetics, being aggressive in comorbid management such as blood pressure optimisation, and shortening tourniquet time in knee replacements.

It is possible that the patient with kidney failure (Fig. 9) could have been missed, because they do not have diabetes and it was a low-risk procedure. However, the patient suffered post-operative kidney failure and POP XGBoost predicted it with 84% confidence. High creatinine and comorbidity burden are the most significant contributions. The high creatinine confirm that this patient likely has impaired renal function, and the prediction could lead to pre-surgical intervention including more intensive management of medications, ensuring the patient is well hydrated, selecting more appropriate anaesthesia type, and optimisation and monitoring of renal perfusion.

Another case study is LOS. The patient underwent a knee-replacement procedure, which is usually a medium LOS. However, POP XGBoost correctly identified this patient as having a high LOS (above five nights) and the SHAP plot (Fig. 10) provides visibility into the reasons. The most significant indicators are comorbidities, a high number of prescribed medications and the procedure itself. As a result of the prediction and indicators, the patient could be booked in for a longer stay and more intensive management.

Most of the studies reviewed, consider interpretability of models to be important for clinical practice, chose algorithms that support it [15] and additionally investigated and reported interpretability results [5, 13, 14, 21, 25, 41, 42]. Lee et al. [42] used a GAM-NN for the benefit of neural networks and the interpretability of GAMs–there is a neural network for each input feature (or group of features), and they are linearly combined for classification.

However, most studies did not consider which features contributed to specific predictions. Bihorac et al. [5] used an approach, where the feature importance was “based on how different she or he is from the patient with an ‘average’ risk”. The reason for the prediction must be inferred indirectly, but the method could be applied to any model. Rajkomar et al. [17] used deep learning neural networks, where interpretability is more of a challenge. They showed a proof-of-concept of how it can be done. Active research is taking place to improve interpretability of deep learning models [43]. The SHAP plots that were demonstrated here, can be used with any model.

### Limitations

The dataset was relatively small for this type of algorithm. For context, other studies cited in this paper range between 51,457 patients [16] to 99,755 [15] admissions and [17] 216,221 admission. We expect the performance to improve with more data, particularly for specific complications, readmission and mortality, as there were very few positive examples to learn from in our study.

In addition to the small dataset, a possible contributor to rare cases is missing or incorrect classifications. For example, ICD codes, which represented complications, are known to be incomplete. The outcomes may be captured in unstructured data, such as patient notes, but these data were unavailable.

The booked procedure is an important factor for predicting risk, according to both clinical practice and the models’ feature importance. However, the booked procedure was not explicitly labelled and was therefore estimated (see “Pre-processing” section), resulting in errors that were difficult to quantify.

Data for patient height and weight were sparse, but these fields are considered to be important patient health factors. Likewise, there were many cases of missing medication therapeutic class, leading to information loss when grouping medications by this variable. Obtaining additional data in these respects is likely to improve performance.

The dataset did not extend beyond discharge, restricting mortality to in-patient mortality. In comparison, most risk calculators predict mortality at various stages after discharge such as 30-day and 60-day mortality. This is clinically important and there would be more examples which would improve the model.

To the authors’ knowledge, there were no shifts to clinical practice over the data collection period (5 years, from 2015 to 2020). However, it is possible that there were subtle changes that would influence the results, in particular the length-of-stay.

### Future work

In future, well-known applied ML techniques for medical risk prediction could be used to improve the initial results; for example, class balancing and model ensembling [44] and data augmentation [18]. There is also scope to explore alternative feature engineering, such as using additional derived features regarding previous admissions, other encoding methods for categorical variables, learning a lower dimensional space for categorical features using decision trees [5], and including additional categories for tests and medications that were ignored in this study.

Another major area of interest is continual risk assessment throughout the admission, including in the post-operative period up until discharge. Only a few related studies considered risk assessment after surgery [17, 21, 42]. It is important because decisions are made throughout the admission and post-surgical care also has the potential to help avoid complications, readmission and mortality.

In future work, the length-of-stay could be converted to an assessment of ‘prolonged’ relative to expectations for specific procedures for additional clinical and operational benefits (“LOS prediction” section). Also, there is a possibility of including the type of procedure as a variable when segmenting LOS to derive the predicted ranges.

A key part of our method was to encapsulate clinical expertise by feature engineering patient-risk and procedure-risk, derived in a manual process. It would be interesting to learn those features in an additional pre-processing step with the potential benefits of time-saving, adaptability and accuracy. One possibility for patient-risk is to use predicted ASA (as done in [45]), provided that ASA targets are available in the training data.

Likewise, the patient’s disease state could be learnt from other variables such as lab results, thus augmenting the use of ICD codes alone; which are subject to human error and can be incomplete, reducing training quality.

This paper is focussed on elective surgery, as described in “Study design” section. Risk prediction is also beneficial for emergency cases; for planning post-surgical care and assisting with selection of intra-operative monitoring. Our method could be tested in this setting.

## Conclusions

In this study, we developed novel algorithms (POP) that exploit tabular EHR data to predict surgical patient outcomes. The algorithms were effective for post-operative complications and LOS in this patient population, but ineffective for predicting readmission and mortality due to extremely rare cases. The results reinforce the importance of using metrics that are suitable for rare cases, which is uncommon in other surgical risk prediction studies. A larger study is justified to improve the algorithms in better predicting complications and length of hospital stay. A larger dataset may also improve the prediction of readmissions and mortality, which were extremely rare. Together with interpretable feature importance plots, surgical risk predictions provide clinically relevant information, that may help to mitigate risks and improve patient outcomes.

### Supplementary Information

**Supplementary material 1.**

## Data Availability

The datasets generated and/or analysed during the current study are not publicly available as there was no data sharing as part of the ethics approval (and raw data is potentially re-identifiable) but are available from the corresponding author on reasonable request.

## Footnotes

1 False Positive Rate is FP/(FP+TN). If FP is high, but TN is very large, the denominator remains high and the rate low.
