# Supplementary material for: Development and validation of ‘Patient Optimizer’ (POP) algorithms for predicting surgical risk with machine learning
Source: BMC Med Inform Decis Mak. 2024 Mar 11;24:70. doi: 10.1186/s12911-024-02463-w (PMC10929113; doi:10.1186/s12911-024-02463-w)
Supplement: Supplementary file 1 — Supplementary material 1. [file 12911_2024_2463_MOESM1_ESM.pdf]

Supplementary Section of Development and Validation of 'Patient Optimizer' (POP) Algorithms for Predicting Surgical Risk with Machine Learning

1 Pre-processing: dimensionality reduction

1.1 'Other' category

Very infrequent categories are grouped into an 'other' bucket. A 'very low' threshold of 10 was chosen based on visual inspection of the frequency histograms, to represent the long tail of numerous categories into one category bucket.

1.2 Reductions

Pathology results are reduced using Max and Min operators, as the extreme values are considered to be the most clinically significant. Demographic and procedure-related numerical fields are reduced with Mean, and if they are not expected to change, the first instance is used. One exception is BMI, where Max is considered more meaningful to the outcome. All the reductions are shown in Table 1.

1.3 Class imbalance

Class balancing was achieved by applying a higher weight to under-represented classes for both model types. In logistic regression, we used the 'class_weight' parameter as 'balanced', and in XGBoost 'scale_pos_weight' was set to the inverse of class proportions in the training data.

Table 1 Reduction methods used when joining one-to-many relationships

Field	Function
Age	Mean
BMI	Max
Height	Mean
Weight	Mean
Gender	First
Pathology tests	Min/Max
Medication	Sum
Length-of-stay (hours)	Max
Unplanned 30-day readmission (boolean)	Or
Discharge deceased (boolean)	Or
Discharge destination	First

2 Hyperparameter tuning

We used Optuna for hyperparameter tuning, which utilises the TPESample algorithm and Hyperband pruner. We used a subset of the hyperparameters that made a difference empirically in preliminary runs with the baseline model: alpha, number of estimators, class balance and maximum depths.
